# Effects of Personalized Cognitive Training with the Machine Learning Algorithm on Neural Efficiency in Healthy Younger Adults

**DOI:** 10.3390/ijerph192013044

**Published:** 2022-10-11

**Authors:** Yu Jin Jeun, Yunyoung Nam, Seong A Lee, Jin-Hyuck Park

**Affiliations:** 1 Department of ICT Convergence, Graduate School of Soonchunhyang University, Asan 31538, Korea; 2 Department of Computer Science, Engineering Soonchunhyang University, Asan 31538, Korea; 3 Department of Occupational Therapy, Soonchunhyang University, Asan 31538, Korea

**Keywords:** prefrontal cortex, neural efficiency, personalization, cognitive training

## Abstract

To date, neural efficiency, an ability to economically utilize mental resources, has not been investigated after cognitive training. The purpose of this study was to provide customized cognitive training and confirm its effect on neural efficiency by investigating prefrontal cortex (PFC) activity using functional near-infrared spectroscopy (fNIRS). Before training, a prediction algorithm based on the PFC activity with logistic regression was used to predict the customized difficulty level with 86% accuracy by collecting data when subjects performed four kinds of cognitive tasks. In the next step, the intervention study was designed using one pre-posttest group. Thirteen healthy adults participated in the virtual reality (VR)-based spatial cognitive training, which was conducted four times a week for 30 min for three weeks with customized difficulty levels for each session. To measure its effect, the trail-making test (TMT) and hemodynamic responses were measured for executive function and PFC activity. During the training, VR-based spatial cognitive performance was improved, and hemodynamic values were gradually increased as the training sessions progressed. In addition, after the training, the performance on the trail-making task (TMT) demonstrated a statistically significant improvement, and there was a statistically significant decrease in the PFC activity. The improved performance on the TMT coupled with the decreased PFC activity could be regarded as training-induced neural efficiency. These results suggested that personalized cognitive training could be effective in improving executive function and neural efficiency.

## 1. Introduction

The human brain requires continuous oxidative metabolism to maintain its function [1]. Accordingly, when a specific brain region is activated, cerebral blood flow in the region increases rapidly [1]. Therefore, it has become possible to observe changes in the brain using non-invasive methods through brain imaging equipment by utilizing these characteristics [2]. In particular, thanks to the advancement of brain imaging technology, it was found that the prefrontal cortex (PFC) is responsible for higher cognition, and this can be confirmed by investigating its activity during cognitive tasks [3].

Functional near-infrared spectroscopy (fNIRS), one of the brain imaging devices, can observe brain activity through changes in the hemoglobin level in a specific area of the brain. Therefore, fNIRS is highly correlated with functional magnetic resonance imaging (fMRI) and blood oxygen level-dependent responses (BOLDs). Moreover, it can measure while moving, is safe, and has a high temporal resolution, which are advantages compared to fMRI [4,5]. Therefore, fNIRS has been actively used in cognitive research areas [6,7,8,9,10,11].

In previous studies on fNIRS, cognitive training was provided at a subject-specific difficulty level based on the subject’s brain activity [6,7,8,9,10,11]. Specifically, when cognitive training at a difficulty level suitable to a subject is provided, they can maintain concentration longer and enjoy high learning effects [12]. Therefore, it is necessary to continuously monitor the subject’s training performance and concentration in hemodynamic responses in regions of interest [13].

In clinical settings, conventional cognitive training is performed through paper-and-pencil tasks or computerized programs, and the subject’s performance is measured. However, since objective indicators, such as the subject’s brain activity, are not measured during conventional cognitive training, clinicians increase the level of difficulty based on the specific success rate of the subject’s performance [14]. However, since the subject’s performance could be affected by confounding factors, such as his or her mood status [15], it is necessary to monitor the subject’s brain activity for personalized and graded cognitive training continuously. Indeed, a previous study confirmed the possibility of implementing personalized cognitive training by providing a subject-personalized level of difficulty based on brain signal-derived neuro-feedback using fNIRS [16]. However, this study presented only changes in the subject’s cognitive performance and brain activity during the training. Similarly, although several studies investigated the effects of cognitive training by measuring brain activity with brain imaging techniques [6,7,8,9,10], they did not confirm its clinical effects on brain plasticity after the training. Thus, in these studies, training-induced neural efficiency was not observed. Neural efficiency could be regarded as decreased brain activity coupled with higher cognitive performance. In other words, neural efficiency refers to high cognitive achievement with a low amount of brain effort [17]. This could be attributed to the fact that most of the training was conducted only for a short period of time in previous studies [6,7,8,9,10].

Moreover, in a previous study, only the left dorsolateral PFC was used as a variable to adjust the difficulty level instead of investigating optimized variables using overall PFC areas related to complex skills. Secondly, it did not present the prediction accuracy for the difficulty classification by exploring various prediction methods, which could affect the effects of the training [16].

Therefore, the purpose of this study was to collect the PFC activity during cognitive tasks using fNIRS and establish an algorithm that could predict the performance level based on the PFC activity. This study also aimed to investigate the effects of personalized cognitive training with the algorithm on neural efficiency. We hypothesized that personalized cognitive training with the algorithm could induce neural efficiency in healthy subjects.

## 2. Materials and Methods

### 2.1. Design

**Figure 1:**
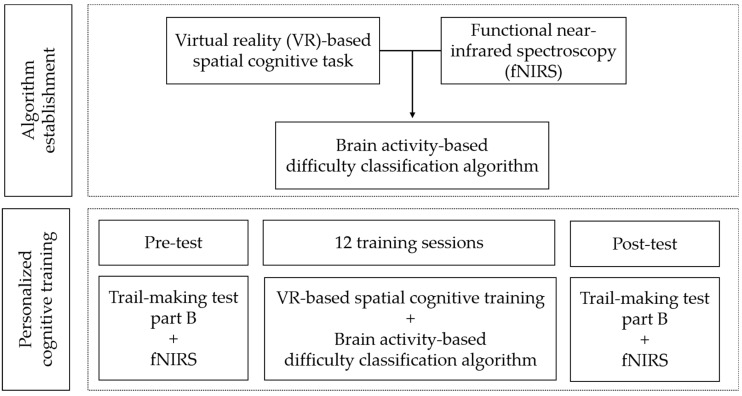
Flow chart for the overall study.

This study measured PFC activity using fNIRS when performing cognitive tasks to establish the algorithm with the highest accuracy by comparing and analyzing various algorithms that could predict the subject’s performance level. Afterwards, this study investigated the effects of personalized cognitive training with the algorithm on neural efficiency in the PFC with the one-group pre-posttest design (Figure 1).

### 2.2. Participants

Sixty subjects were recruited from a local college in Asan-si, South Korea, and were randomly assigned to the data collection group or the training group. Among them, 45 were allocated to the data collection group for the algorithm, and 15 were assigned to the training group using convenience sampling. However, in the training group, 2 subjects were excluded because one had to be isolated due to the coronavirus disease-19 situation, and the other subject’s data was not saved; thus, the data of 13 subjects were finally analyzed. The inclusion criteria were (a) adults over 20 years old, (b) healthy conditions without visual or auditory impairments, (c) intact global cognitive function confirmed with a score ≥ 24 on the Korean version of the mini-mental status examination, and (d) people who did not have barriers to using a computer. The exclusion criteria were as follows: (a) experience of any pharmacological or non-pharmacological cognitive treatment within the past three months, (b) the presence of neurological or psychiatric diseases, and (c) the presence of a disease requiring medical care.

This study was approved by the local Institute of Review Board (No. 202104-SB-031), and written consent was obtained from all subjects in accordance with the Declaration of Helsinki. All experiments were conducted in a laboratory to provide the same experimental environment. Due to the coronavirus disease-19 (COVID-19) pandemic, all subjects wore masks the same way.

### 2.3. Machine Learning Algorithm

In order to establish the optimized algorithm, some machine learning algorithms for difficulty-level classifications using datasets were compared. Subjects for the data collection group were 45 healthy adults in their twenties, and they performed the verbal digit span task mainly related to the PFC, while their PFC activity was measured using fNIRS [18,19,20]. A total of 180 fNIRS data was collected, and then 159 data were analyzed after excluding the results with omissions, sensor outliers, and result outlier values during the pre-processing process.

This study used the support vector machine (SVM), K-nearest neighbor (KNN), decision tree (DT), and logistic regression machine learning algorithms using the Scikit-Learn library [21] to establish the optimized algorithm. The first step was the pre-processing phase which standardized the raw data of the verbal digit span task and the oxy-hemoglobin (HbO) values in the PFC. The second step was to randomly divide the standardized data into a training dataset and a test dataset at a ratio of 7:3. The third step was the learning process of the machine learning model to classify two difficulty statuses (high and low). F regression analysis was used to extract a feature which could accurately classify the difficulty levels.

### 2.4. Intervention

Virtual reality (VR)-based spatial cognitive tasks, run using a desktop computer, were implemented for 12 sessions (40 min a session for 3 weeks). Before the training, each subject was provided practice time to become familiar with the VR task. The VR-based spatial cognitive task was a computerized training program designed to improve spatial memory and was implemented in a 3D form and developed using the Unity game engine [22]. The VR environment used walls and windows for the indoors and mountains and trees for the outdoors as designs to limit boundaries. In this environment, no object that could become a landmark in the background was found, and there was only a background for zone limitation. The subjects used a keyboard and mouse to move freely in the VR environment. In this task, subjects were immersed in a VR environment with boundaries. The subjects’ initial locations were randomly designated in the environment, and then they were asked to look around. They were asked to move towards a destination represented with twinkling stars. Once they arrived, another destination was again allocated at a different location from the first. They were instructed to move to reach that point, resulting in the subject being moved to a different location. Once they arrived at the second destination, the subjects were moved back to the initial location. The number of destinations depended on the difficulty level. The subjects needed to find three destinations in the low difficulty level and four destinations in the high difficulty level. The subjects were instructed to move to the destinations in order and press a mouse without a destination indication. In other words, the subjects had to estimate the destinations using indoor and outdoor cues. All performances in all sessions were recorded using the Euclidean distance between the actual locations and the estimated locations.

During each session, the subjects were equipped with fNIRS to measure their PFC activity for the training difficulty classification. According to the subjects’ PFC activity, the VR-based spatial cognitive task was adjusted to the high or low difficulty level after each trial.

One occupational therapist with 3 years of clinical experience conducted all sessions. The therapist sufficiently practiced the process of the personalized difficulty levels using the subject’s brain activity.

### 2.5. Measurements

The trail-making test part B (TMT-B), an executive function assessment, was used to evaluate the neural efficiency in the PFC with the fNIRS device. The TMT is an assessment that evaluates attention, working memory, and cognitive flexibility tests [23]. The TMT-B requires connecting randomly listed numbers and alphabets alternatively as quickly as possible. Randomly listed numbers were from 1 to 13, and randomly arranged alphabets were from A to L. If a subject achieved three or more errors during the test, the test was terminated. The TMT-B was used to evaluate the shift attention, which is related to the executive function depending on the PFC [24]. Its performance was the reaction time taken to complete the test, and a lower score meant higher executive function.

**Figure 2:**
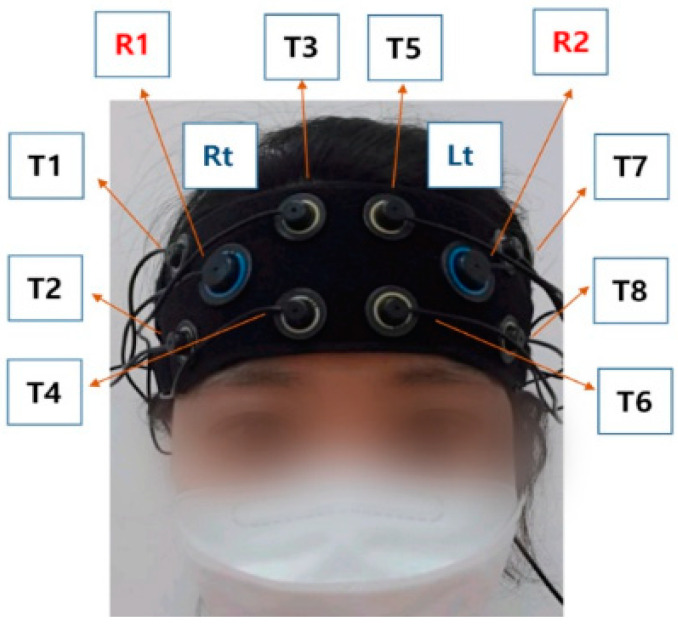
Channel locations measured with OctaMon (fNIRS). R, light source; T light detector.

The PFC activity during the TMT-B was measured using the OctaMon fNIRS device (Artinis, The Netherlands). This device has 8 channels (2 × 4 channels), and the combination of a light source and detector records the concentration levels of HbO and deoxidized hemoglobin (HHB) in the PFC in real-time using wavelengths of 760 nm and 850 nm (Figure 2). This study set the distance between the light source and the detector to 35 mm to measure the changes in HbO in the PFC [25]. The light source and detector pairs were placed over the left (Fp1) and right (Fp2) frontal cortex regions according to the modified international electroencephalography (EEG) 10–20 system, targeting the left and right PFC. The subjects wore the device after sitting on a chair and resting for 5 min to stabilize blood flow. The subjects were asked to wipe their forehead with alcohol swabs to prevent their hair from falling to their forehead and to restrict their head movements during the measurement process.

### 2.6. fNIRS Data

Data extracted from the eight channels of the fNIRS were pre-processed using Oxysoft 3.0.52 (Artinis, The Netherlands). They were analyzed after removing the noise caused by spikes, high-frequency noise, and heart rates by applying a low pass filter with a cutoff frequency of 0.5 Hz to the measured signal [26,27]. All fNIRS data were sampled after removing the 4 s at the front due to the time delay of the hemodynamic response with a frequency of 10 Hz [28,29].

### 2.7. Statistical Analysis

All statistical analyses were conducted using the SPSS 22.0 version (IBM corp., Armonk, NY, USA). Since the normal distribution was not satisfied in the Shapiro-Wilk test, the Wilcoxon signed-rank test was used to check whether there was a significant difference before and after the intervention, and the statistical significance was set at *p* < 0.05.

## 3. Results

### 3.1. General Characteristics of Subjects

Two subjects in the training group dropped out; therefore, the data of 13 subjects in the training group were finally analyzed. The general characteristics of the subjects in both the algorithm group and the training group are presented in Table 1. All subjects were in their twenties, and unbalanced sex ratios were found (algorithm group: 35 females (77.7%), 10 males (22.3%); training group: 12 females (92.3%), 1 male (7.69%)). There was no additional information on general characteristics, such as occupation, as all subjects were college students.

### 3.2. Algorithm

Among the eight measured channels, the channels which represented the ventrolateral PFC areas were identified to be the most accurate for the difficulty classification, which meant that the two channels’ data were only used for selecting the difficulty level in the algorithm. Four machine learning algorithms used the activity in the ventrolateral PFC as a variable to classify the difficulty 10 times, and the classification accuracy was recorded at an average of 10 times. The classification accuracy was performed by one researcher using Python and supervised by another researcher. Among the machine learning algorithms, the logistic regression-based algorithm showed the highest accuracy (86.24%) (Table 2). Based on the algorithm, subjects in the training group performed personalized cognitive training for 12 sessions. During each session, the training difficulty was selected by inputting each subject’s ventrolateral PFC activity measured during the training into the algorithm

### 3.3. Virtual Reality-Based Spatial Cognitive Task Performance According to the Training Sessions

**Figure 3:**
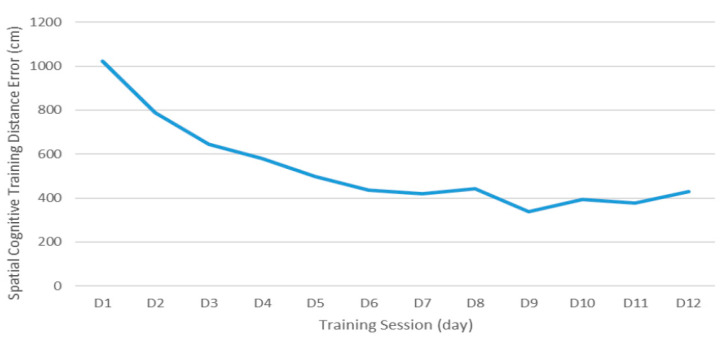
Change in performance on the spatial cognitive task during training.

The error between the destination location and the estimated location tended to decrease over the 12 training sessions (Figure 3). Although there was a slight difference depending on the session, the error generally decreased. Specifically, the performance error continuously decreased every session and remained at a similar level after the 8th session.

### 3.4. PFC Activity According to the Training Session

**Figure 4:**
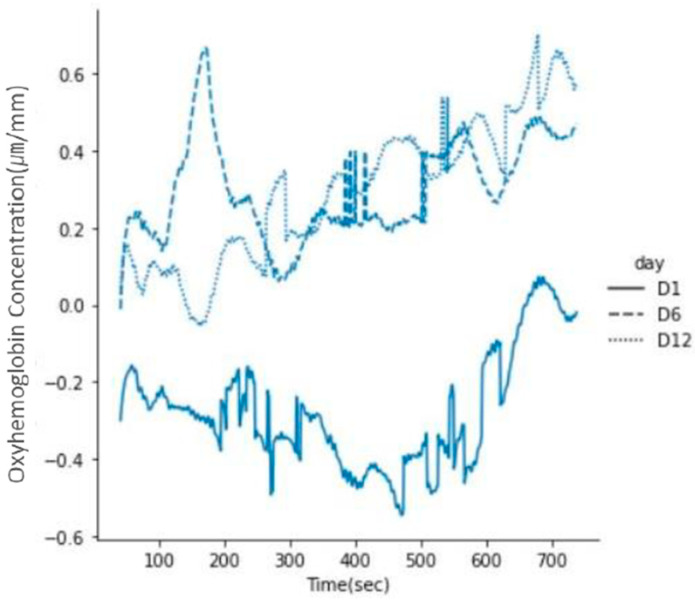
Change in the PFC activity during the training sessions.

The mean of the HbO in the PFC was compared by dividing it according to the three training sessions (1st, 6th, and 12th sessions). As a result, the mean of the HbO in the PFC tended to improve from the 1st session to the 6th session, and it was constant in the last 12th session without a sudden change. In other words, the PFC activity at the 6th and 12th sessions were higher than in the 1st session (Figure 4).

### 3.5. Neural Efficiency

The TMT-B performance was significantly improved after 12 training sessions (z = −3.18, *p* = 0.001), which suggested that personalized cognitive training enhanced executive function. On the other hand, the mean of the HbO during the TMT-B considerably decreased in the overall PFC areas after 12 training sessions. Specifically, the mean of the HbO significantly decreased in the overall PFC (z = −2.341, *p* = 0.019), the left PFC (z = −2.621, *p* = 0.009), and the ventrolateral PFC (z = −2.062, *p* = 0.039) (Table 3).

## 4. Discussion

This study aimed to investigate the effects of personalized cognitive training with the machine learning algorithm on neural efficiency in healthy adults after 12 training sessions. It found that personalized cognitive training enhanced executive function and decreased PFC activity during executive function testing, which is consistent with previous studies [30,31].

In this study, the algorithm which classified the training difficulty levels was established using the PFC activity-based machine learning methods. Specifically, the activity from the ventrolateral PFC was found to be the best indicator to classify the difficulty levels accurately. The ventrolateral PFC area is well-known as related to allocentric spatial cognition with the medial temporal lobe regions [32,33]. Since the VR-based spatial cognitive task requires an allocentric spatial strategy to find the destinations, the ventrolateral PFC was chosen as the feature for establishing the algorithm for the difficulty level, which is consistent with previous studies. In other words, the ventrolateral PFC activity affected the algorithm more than the other regions of the PFC.

Over the training sessions, it was confirmed that more repetitive training sessions could improve performance. Specifically, the performance error continuously decreased every session and remained at a similar level after the 8th session, which could be because of the ceiling effect. Specifically, since the subjects performed cognitive training with only two difficulty levels, the algorithm could demonstrate that according to the subject’s brain activity, subjects maintained their high performance without a higher level of cognitive challenge [34]. Moreover, personalized cognitive training increased PFC activity. In particular, it was found that the PFC activity improved over training sessions from the initial session, and it increased in the 12th session compared to the 6th session. This trend is partially consistent with the results of a previous study which reported that the hemodynamic changes in the PFC were not only linear but also non-linear after training with a spatial working memory task [35]. On the other hand, the PFC activity was relatively low in the first training session compared to the other sessions because proper neural recruitment was not executed properly in the first session to perform the task. This result is consistent with a previous study [36], which showed that individuals with higher cognitive function showed higher brain activity than people with lower cognitive function. The PFC activity showed a trend that increased as the training was repeated from the 1st session to the 6th session, and the changes in the PFC activity occurred with a gentle and uniform slope moving to the 12th session. This result could be interpreted as the neural recruitments gradually increased to respond appropriately to the cognitive challenges [35]. These findings suggested that personalized cognitive training could enhance spatial memory and neural recruitment.

Repetitive cognitive exercises could promote neuroplasticity, resulting in improvement in both target cognitive function and non-targeted cognitive function. Unfortunately, to date, improvements in cognitive function are mainly investigated using cognitive performance rather than observing both cognitive performance and neuroplasticity [37]. In contrast, this study confirmed the effect of cognitive training in terms of neuroplasticity through an improvement in neural recruitment as well as cognitive performance. Despite this, the superiority of personalized training was not confirmed by comparing it with the previous one; our new approach was also found to be effective, which is the clinical implication of this study.

After training, the subjects showed decreased PFC activity during executive functioning, coupled with an improvement in executive function. In other words, the subjects achieved better executive performance with a lower amount of brain effort. Decreased activity in the PFC with better performance on cognitive assessments could be considered training-induced neural efficiency [38]. Indeed, previous studies consistently indicated decreased brain activity coupled with improved cognitive function as a training-induced clinical effect, supporting our findings [39,40]. In particular, in this study, the activity on the left side of the PFC significantly decreased compared to the right side of the PFC. This could be attributed to the characteristics of the TMT-B. The TMT-B mainly examines the executive function, which mainly depends on the PFC [31]. In a previous study, the left PFC is one of the brain regions which could be highly activated during the TMT-B [31], which supports the findings of this study.

One crucial clinical implication of this study is that increased neural efficiency in the PFC coupled with improved cognitive function was found through a relatively short period of personalized cognitive training in younger adults without cognitive impairments. Cognitive training with a level of difficulty adjusted in a conventional way based on a success rate has also been identified as effective in improving cognitive function, but it is difficult to provide individually tailored training. With the advancement of brain imaging technology, various brain imaging devices have been actively adopted in cognitive rehabilitation fields [6,7,8,9,10,11]. Regardless, the algorithm was established with a small number of subjects, and the personalized cognitive training was found to be sufficient for inducing neural efficiency in the PFC, suggesting that this training could be clinically beneficial. This result suggests that this kind of cognitive training could be clinically beneficial for not only individuals without cognitive impairment but also patients with cognitive impairment. Nevertheless, since neural efficiency could depend on clinical symptoms and the severity of cognitive impairment, it is necessary to investigate the brain areas sensitive to cognitive training and the effects of cognitive training using algorithms based on their activity [41].

This study, however, has some limitations. Firstly, this study could not provide training with a wide range of difficulties as the algorithm could only classify the difficulty as high or low. Since no algorithm with various levels of difficulty was established (due to the small number of subjects), it is necessary to further subdivide the difficulty in future algorithms. Secondly, an algorithm using more machine learning models that consider the characteristics of the collected data was not established. Indeed, the time series and slope of the brain activity values might be analyzed using long short-term memory models or images by converting the overall brain activity data into two dimensions [42]. Thirdly, since the design of this study was a one-group pre-test-post-test design without a control group, the effects of the personalized cognitive training need to be interpreted with caution. Fourthly, although executive function involves various sub-elements, such as mental flexibility and attention shifting, this study solely assessed the executive function using the TMT-B. Therefore, it is difficult to conclude that neural efficiency has occurred in all executive functions. Nevertheless, the TMT-B is well-known as a representative assessment for executive function. Fifthly, the small sample size limits the ability to formulate definitive conclusions about the efficacy of personalized cognitive training. In addition, provided that cognitive training has been widely used for people with cognitive impairments, future studies need to investigate its efficacy on subjects with cognitive impairments. Finally, all subjects wore masks during all the experimental procedures because the experiment was conducted during the COVID-19 pandemic. Wearing a mask is not a common training condition, and wearing a mask could influence the measurement of the HbO in this study. However, it is believed that there is no difficulty in confirming the effects of the training on brain activity because the mask was worn the same when the dataset was collected to test the algorithm.

## 5. Conclusions

The findings of this study have demonstrated that personalized cognitive training with the machine learning algorithm could be effective in improving cognitive function and neural efficiency in the PFC. Considering that the training difficulty provided by the brain activity as objective, personalized training has rarely been investigated, the current findings have significant clinical implications. This study also sheds new light on the potential application of an fNIRS device as a tool to examine the effects of cognitive training. In the future, the effects of personalized cognitive training with diverse machine learning algorithms need to be investigated using an fNIRS device in clinical populations with cognitive impairments, such as mild cognitive impairment.

## Figures and Tables

**Table 1:** General characteristics of the participants (*n* = 58).

		Algorithm Group (*n* = 45)		Training Group (*n* = 13)	
		*n*	%	*n*	%
Sex	Females	35	22.3	12	92.3
	Males	10	77.7	1	7.69
Age (years)	Mean (SD)	20 (1.26)		21.9 (2.29)	
Dominant hand	Rt	44	97.8	13	100.0
	Lt	1	0.02	0	0

**Table 2:** Classification accuracy of algorithms according to the machine learning models.

	SVM	KNN	DT	Logistic Regression
Accuracy (%)	85.62	82.49	78.93	86.24

SVM, Support vector machine; KNN, K-nearest neighbor; DT, decision tree.

**Table 3:** TMT-B performance and the PFC activity before and after 12 training sessions.

	Mean (SD)		z	*p*
	Pre-Test	Post-Test		
TMT-B reaction time (s)	76.33 (20.57)	51.16 (9.6)	−3.180	0.001 *
HbO (μM/mm)				
Overall PFC	1.15 (0.63)	0.23 (0.85)	−2.341	0.019 *
Right PFC	1.09 (0.75)	0.39 (0.77)	−1.852	0.064
Left PFC	1.21 (0.86)	0.08 (1.04)	−2.621	0.009 *
Dorsolateral PFC	0.96 (0.83)	0.33 (1.09)	−1.852	0.064
Ventrolateral PFC	0.99 (0.78)	0.18 (0.72)	−2.062	0.039 *

* *p* < 0.05, SD, Standard deviation; PFC, prefrontal cortex.

## Data Availability

The data presented in this study are available upon request from the corresponding author. The data are not publicly available because they are part of an ongoing project.
